# Trends in breast cancer incidence in Hong Kong between 1973 and 1999: an age-period-cohort analysis

**DOI:** 10.1038/sj.bjc.6600583

**Published:** 2002-10-21

**Authors:** G M Leung, T Q Thach, T-H Lam, A J Hedley, W Foo, R Fielding, P S F Yip, E M C Lau, C-M Wong

**Affiliations:** Department of Community Medicine, University of Hong Kong, 5/F, Academic & Administration Block, Faculty of Medicine Building, 21 Sassoon Road, Pokfulam, Hong Kong, China; Hong Kong Cancer Registry, Hospital Authority, c/o Department of Clinical Oncology, Queen Elizabeth Hospital, 30 Gascoigne Road, Kowloon, Hong Kong, China; Department of Statistics and Actuarial Science, University of Hong Kong, Pokfulam Road, Hong Kong, China; Department of Community and Family Medicine, Chinese University of Hong Kong, Prince of Wales Hospital, Shatin, Hong Kong, China; Cancer Expert Working Groups (Prevention and Screening; Data and Priorities), Cancer Coordinating Committee, Health, Welfare and Food Bureau, Government of the Hong Kong Special Administrative Region, Murray Building, 3 Garden Road, Central, Hong Kong, China

**Keywords:** breast cancer, incidence, age-period-cohort, Poisson distribution, Hong Kong

## Abstract

Hong Kong has the highest breast cancer incidence in Asia and studying secular changes in its rates may lead to hypotheses regarding disease aetiology and also predictions of future trends for China. We examined statistics from the Hong Kong Cancer Registry based on 26 566 cases of invasive breast cancer from 1973 to 1999. The trends in breast cancer incidence were studied using log-linear longitudinal models. We further analysed the independent effects of chronological age, time period and birth cohort on incidence trends using age-period-cohort modelling. The average annual per cent change of the age-standardised incidence was 3.6% during 1973–1999. Age-period-cohort modelling indicated the incidence development was predominantly a cohort effect, where the rise in relative risk was seemingly linear in successive birth cohorts, showing a 2–3-fold difference when comparing women born in the 1960's with those born around 1900. Our results suggest that direct and indirect consequences of westernisation may have been responsible for most of the observed increase in breast cancer incidence. As China moves towards a more westernised way of life, we can expect an emerging epidemic of breast cancer as Hong Kong's experience has demonstrated.

*British Journal of Cancer* (2002) **87**, 982–988. doi:10.1038/sj.bjc.6600583 www.bjcancer.com

© 2002 Cancer Research UK

## 

Cancer incidence should be assessed separately in different populations, as it may differ substantially from one population to another in ways that are difficult to predict. Thus, the US and UK reported age-standardised rates of 90.7 and 68.8 per 100 000 women compared to 34.0 for Hong Kong and 11.2–26.5 per 100 000 for China (Qidong, Shanghai and Tianjin) during 1988–1992 (Parkin *et al*, 1997).

Our primary aim is to determine if the difference in breast cancer incidence between Caucasian women and their Chinese counterparts can be explained by lifestyle and behavioural factors as indicated by birth cohort effects (Yu *et al*, 1991). Will breast cancer incidence in the East follow the upward trend observed in the West as it adopts an increasingly Western lifestyle?

China, with 20% of the world's female population, needs direct evidence of the eventual effects of westernisation on locally specific morbidity and mortality associated with breast cancer. However, China only started to adopt Western lifestyle practices recently and therefore it is too early for the full effects to be seen. Studying the changes in Hong Kong's secular breast cancer rates may reveal what is likely forthcoming for China. The present paper describes an analysis of longitudinal breast cancer incidence rates in Hong Kong, in particular distinguishing time period and birth cohort as determinants of secular trends.

## METHODS

### Sources of data

Data on breast cancer incidence were obtained from the Hong Kong Cancer Registry. Details of the history, objectives, logistics and registration coverage of the Cancer Registry are documented elsewhere (Hong Kong Cancer Registry, 1999). Briefly, the Hong Kong Cancer Registry is a population-based registry covering the entire resident population of Hong Kong. Information on breast cancer cases were collected from both the private and public service sectors (mainly through departments of clinical and radiation oncology and histopathology), and from the Government's Births, Deaths and Marriages Registry, as well as voluntary notification from all medical practitioners. The completeness and quality of the data has been reported to be good, especially in the last 15 years (Hong Kong Cancer Registry, 1999), and the Hong Kong Cancer Registry is an accredited member of the International Association of Cancer Registries (Parkin *et al*, 1997). Data on mid-year population statistics were derived from the Government's Census and Statistics Department.

The present analyses were based on 26 479 cases (out of a total of 26 566 cases where the age at diagnosis was unknown in 87 cases) of invasive breast cancer (International Classification of Diseases 8th edition (ICD-8) and ICD-9 code 174) reported from all medical institutions in Hong Kong during a 27-year period from January 1973 to December 1999.

### Statistical analysis

On the basis of this data set, 3-year age-adjusted incidence rates were calculated by direct standardisation with the World Standard Population for the period from 1973–1975 to 1996–1999 (Parkin *et al*, 1997).

Secular trends in the incidence of breast cancer based on annual data were first examined with a simple log-linear regression model. This model forms the basis for the estimates of the average annual per cent change (AAPC) in rates with time periods. A two-tailed test of statistical significance was applied to the AAPC (Tarone and Chu, 1992). To test for possible non-linear trends, second-order polynomial models including a quadratic trend term were also constructed.

We further analysed the independent effects of chronological age, time period and birth cohort on incidence trends in breast cancer using age-period-cohort modelling. Cases were grouped into 5-year age groups (from 30–34 years to 80–84 years). There were very few cases in the age groups below 30 years or above 85 years and resultant rates were unstable, therefore we omitted these age groups from the analysis. Similarly, the time periods of diagnosis were divided into 5-year intervals from 1975–1979 to 1996–1999. A two-way table of age group by time period was constructed giving a total of 11 age groups, five time periods and 15 synthetic birth cohorts. (Table 1

**Table 1:**
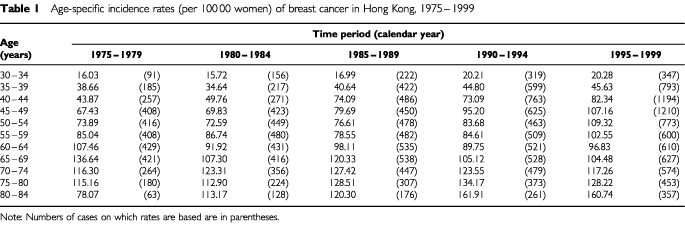
Age-specific incidence rates (per 100 00 women) of breast cancer in Hong Kong, 1975–1999

) With 5-year age and period groups the women contributing to a birth cohort were born within a 10-year period, i.e. the same woman contributing to two adjacent synthetic birth cohorts. The diagonals of the two-way table represent these synthetic birth cohorts with the first cohort corresponding to those aged 80–84 years during 1975–1979 (i.e. born in 1890–1899); the second cohort were aged 75–79 years during 1975–1979 (i.e. born in 1895–1904), and so on. Thus there were 15 such overlapping 10-year birth cohorts, ranging from 1890–1899 to 1960–1969. The usual convention to identify these cohorts is to take the central year of birth from 1895 to 1965.

To obtain the effects of age, period and cohort on breast cancer incidence, models were fitted on the assumption that the number of cases constituted a variable with a Poisson distribution. The incidence rates were assumed to be a multiplicative function of the included model parameters, making the logarithm of the rates an additive function of the parameters (Clayton and Schifflers, 1987a,b; Holford, 1991, 1992).

In the usual notation of an age-period-cohort model, the logarithm of the incidence rate is expressed as a linear combination of the effects of age, period and cohorts:

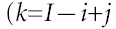

where λ_*ijk*_ denotes the incidence rate in the *i*th age group, *j*th period and *k*th cohort; μ, the intercept; α_*i*_, the effect of the *i*th age group (*i*=1,2,…,*I*); π_*j*_, the effect of the *j*th time period (*j*=1,2,…J); γ_*k*_, the effect of the *k*th birth cohort (*k*=*I*−*i*+*j* when *i*=1,2,…,*I*); and ε_*ijk*_ is the random error term.

 Parameter estimates in the age-period-cohort model were generated using the maximum likelihood method and interpreted as the log of the relative risk, adjusted for the other two factors. In our study, the calendar period 1985–1989 and birth cohort with central year of birth 1925 were adopted as reference categories. A sequence of models was fitted starting with the one-factor age model, progressing to the two-factor age-drift, age-period and age-cohort models, and finally to the full three-factor age-period-cohort model.

In the analyses of age-period-cohort models, a fundamental problem is the linear dependence between age, period and cohort effects, i.e. the estimates of α_*i*_, π_*j*_ and γ_*k*_ are not unique, or identifiable (cohort=period−age). There has been much discussion in the literature about various approaches to overcome this. All such approaches require imposing further restrictions on the full model in order to obtain unique effects. One strategy to solving the non-identifiability problem is to assume the regression coefficients of the first and last periods to be zero, a technique commonly used in several previous studies (Tarone and Chu, 1996; Shahpar and Li, 1999). With this assumption, it was possible to obtain first-order relative risk estimates and associated 95% confidence intervals for the cohort effects (Holford, 1991; Persson *et al*, 1993). Similarly, relative risks for period effects were generated by imposing constraints such that the regression coefficients of the first and last cohorts were assumed to be zero.

The deviance of the model was used to measure the goodness-of-fit. A smaller deviance implies a better fit but attention should be paid also to the degrees of freedom, which is in turn a function of the difference between the number of observations and the number of estimated parameters in a given model. To test for significance of effects between the full three-factor model and nested models (i.e. two-factor age-period and age-cohort models), we compared the difference in deviance between these different models using the F test (Mccullagh and Nelder, 1983).

All analyses were conducted using S-PLUS software version 3.4.

## RESULTS

**Figure 1:**
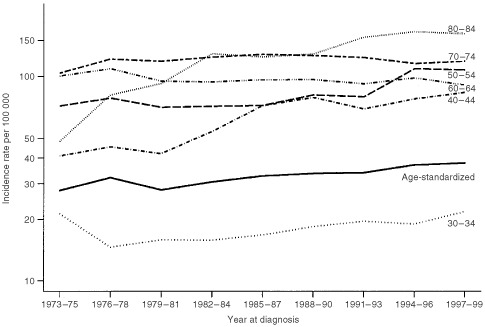
Age-standardised (World Standard Population) and selected age-specific incidence rates for breast cancer by year of diagnosis, 1973–1999

Age-standardised as well as age-specific trends in incidence rates over the entire period of observation were plotted on a logarithmic scale (Figure 1

). The overall age-adjusted rate increased steadily from 27.7 in 1973–1975 to 37.9 per 100 000 women in 1997–1999. This rise was reflected in all age groups, except for those aged 60–69, throughout the 27-year period and especially in the younger age groups. The increase for most age-specific rates and the age-adjusted trend appeared to be accelerated in the latter part of the observation period.

We sought to confirm these observations through visual inspection by calculating the AAPC, assuming the same rate of change during the whole period (Table 2

**Table 2:**
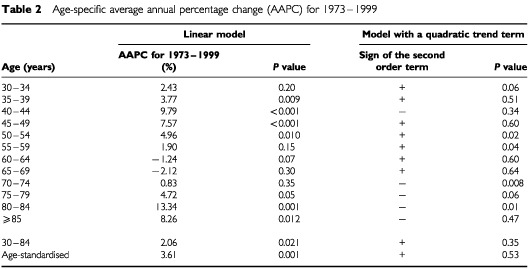
Age-specific average annual percentage change (AAPC) for 1973–1999

). The overall age-standardised incidence increased annually by 3.61% during the 27-year period. A significant change was detected in most age groups, ranging from −2.12% to 13.34%. The most marked changes occurred in the groups below 50 years and above 80 years. For the four oldest age groups, the quadratic trend term was negative, indicating that the rate of increase has slowed down in recent years.

Age-period-cohort models were fitted to the data for 1975 through 1999. Table 3

**Table 3:**
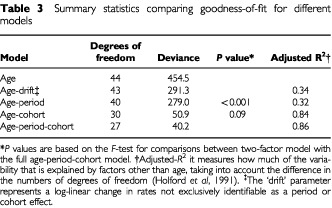
Summary statistics comparing goodness-of-fit for different models

shows the change in deviance, or goodness-of-fit, in the sequential building of the models. Both age-period and age-cohort models significantly improved the fit over the age only and age-drift models. Tests were significant for period effects (change in deviance (CD)=175.54, degrees of freedom (df=4), *P*<0.001) and cohort effects (CD=403.59, df=14, *P*<0.001) after adjustment for each effect and age. Most of the observed age-adjusted changes in incidence rates could be explained by cohort effects (adjusted *R*^2^=0.84) whereas period effects (adjusted *R*^2^=0.32) had a relatively minor contribution (Holford *et al*, 1991). The full model, however, was superior to both the nested age-period and age-cohort models (adjusted *R*^2^=0.86), although there was no statistically demonstrable difference between the two-factor age-cohort model and the full age-period-cohort model (*P*=0.09 by the *F* test). Figure 2

**Figure 2:**
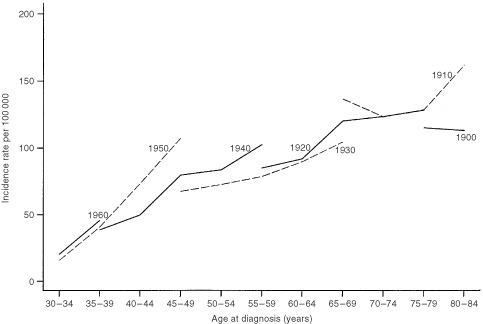
Age-specific incidence of breast cancer by birth cohort

displays the age-specific incidence of breast cancer by birth cohort where the parallelism in the curves confirms the strong cohort effects on overall disease rates (Seow *et al*, 1996).

Relative risks were calculated by time period and birth cohort, based on two separate full age-period-cohort models with cohort and period constraints respectively (Table 4

**Table 4:**
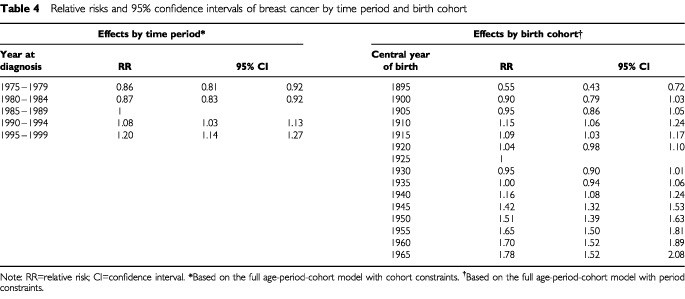
Relative risks and 95% confidence intervals of breast cancer by time period and birth cohort

). There is a clear linear rise starting from the early 1980's where we observed a 38% excess risk of breast cancer in the most recent time period compared to 1980–1984. Figure 3

**Figure 3:**
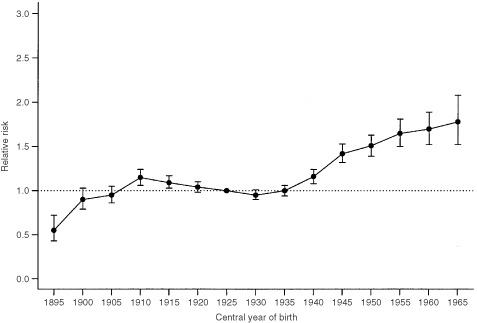
Note: Based on full age-period-cohort model with period constraints. Bars around the point estimate indicate the 95% confidence intervals. Relative risk estimates of incidence of breast cancer by birth cohort

supplements the tabulated relative risks by birth cohort and reveals that there is a general upward trend with succeeding cohorts. The rate of increase appears to be steepest for the earliest few birth cohorts although it should be noted that there were less data available for these groups and data that were recorded could have suffered from under-reporting during the early years of the Hong Kong Cancer Registry.

There were two inflection points of note, namely during the central years of birth of 1910 and 1930. To determine whether the slopes before or after a particular birth cohort were significantly different (i.e. second-order changes), we tested the linear contrast of the form (γ_*i*+1_−γ_*i*_)–(γ_*i*_−γ_*i*−1_), where γ_*i*_ denotes the *i*th birth cohort effect and negative values reflect an increase in the slope. The likelihood ratio statistic was used and it follows an approximate chi-square distribution. This revealed a significant decrease in the birth cohort effect between the 1910 and 1930 birth cohorts (*P*=0.001), followed by a significant increase after the 1930 cohort (*P*<0.001).

Relative risk estimates of the cohort effects were also obtained from the two-factor age-cohort model. The values of the relative risks were almost identical to those from the full model as reported in Table 4. The magnitude of the risk estimates from period effects in the two-factor age-period model were more dissimilar to the corresponding full model, which would be expected given the wide disparity in *R*^2^ between the two models (Table 3), although the direction of change remained the same.

## DISCUSSION

We used the age-period-cohort approach, a statistical modelling procedure, to disentangle the relative contributions of chronological age, time period, and birth cohort on breast cancer incidence in Hong Kong women from 1973 to 1999. By cohort effect, we mean variations in disease rates observed between generations reflecting the different causal factors to which each successive birth cohort is exposed as the environment and society change. Period effects refer to incidence variations due to environmental changes at specific time points (e.g. World Wars I and II).

We found that the overall age-standardised incidence of female breast cancer showed average annual increases of 3.6% between 1973 and 1999. Of note, this increase was most marked in the younger age groups and appeared to accelerate. Whereas most Western countries have reported declining incidences or slowing rates of increase, Hong Kong is just beginning to experience the accelerated increase which Western nations observed 20 years earlier (Wun *et al*, 1995). Analyses using age-period-cohort modelling revealed that the incidence development was mostly explained as a cohort effect, although there was also minor but definite contribution from period effects. The rise in relative risk was apparently linear in successive cohorts, showing a 2–3-fold difference between women born in the 1960's and those born at the turn of the twentieth century.

Bias due to secular improvements in cancer registration is unlikely to explain most of the observed changes. Although notification is voluntary, the comprehensiveness in coverage of the Hong Kong Cancer Registry has been reported to be good especially in the last 15 years. In addition, case ascertainment by histological verification was achieved consistently for more than 80% (95.8% in 1999) of cases. As with most cancer registries, coverage was probably less complete in the 1970's and the early 1980's. This would have led to some of the period effects that were found. The period effects shown in Table 4 reflect changes that affect all age cohorts at a given time and apart from under-reporting in the earliest periods may be due to changes related to health services and breast cancer detection (Rostgaard *et al*, 2001).

It is well known that increased cancer detection activities, notably mammographic screening examinations, may enhance incidence rates (Persson *et al*, 1993; Wun *et al*, 1995; Rostgaard *et al*, 2001). Hong Kong, however, has never had an organised screening programme. Screening has only become more popular since the early 1990's although the precise extent of its population coverage is unclear (Leung *et al*, 2002a). We did not find a change in the slope of the period effects curve (figure not shown) in the last decade as would be expected had screening mammography exerted a population impact, rather the line was almost perfectly linear throughout the 25-year time frame. We also would not expect a near linear increase of cohort effects starting with women born in the 1930's through the most recent cohorts of the 1960's because these latter generations would not have been old enough to be included in the screening target group (Figure 3). The parallelism demonstrated in Figure 2 further suggests a set of exposures that exerts its primary effect early in life before the age of appreciable risk is attained being responsible for the observed outcome, again casting doubt on the contribution from screening interventions on the eventual disease rate (Seow *et al*, 1996). Lastly, the Cancer Registry only included invasive cancers whereas screening has been shown to be most prone to cause artefactual increases in incidence through ductal carcinoma *in situ* diagnoses (Persson *et al*, 1993).

Factors that change from one generation to another need consideration: first, Hong Kong's total fertility rate (TFR) fell from 2.5 children per woman in 1976 to below 1.0 in 1998 (Yip *et al*, 2001). Women born since the 1940's would be responsible for this decrease and they were also the ones experiencing the majority of the cohort effects shown. Although available data preclude a formal correlational analysis, the observed trends support an inverse effect of fertility on breast cancer risk. This probably means that there has been an increase in the proportion of nulliparous women, in addition to a decrease in parity. Second, if obesity is an independent risk, especially in postmenopausal women, as many studies indicate, decreased physical activity as more women enter sedentary jobs from more physically active domestic work would explain some of the observed cohort effect. Occupational activity may have a particular protective effect in women of certain age groups, for instance in the 50–59 age group (i.e. the age of the 1920–1929 cohort in the 1980's) (Moradi *et al*, 1999). Third, the increasing affluence of Hong Kong's population has probably been responsible for shifting the age of menarche earlier (from 12.6 years in 1981 to 12.07 in 2001 (The Family Planning Association of Hong Kong, 1981, 1986, 1991, 1996, 2001)) although it is difficult to validate this ecological observation (Madigan *et al*, 1995). Lastly, although we do not have reliable population-based, longitudinal estimates of age at first pregnancy, we should recognise its potential importance in the observed cohort effect.

It has been reported that the longer women breastfeed the more they are protected against breast cancer (Collaborative Group on Hormonal Factors in Breast Cancer, 2002). We do not have time series data on breastfeeding patterns in Hong Kong although we speculate that breastfeeding was highly prevalent in the 1900's when Hong Kong was still a developing economy and gradually decreased to very low levels until the 1980's as it rapidly transitioned to a developed society, thereby accounting for some of the observed breast cancer incidence increase from cohort effects. Between 1987 and 1997, the ever breastfeeding rate actually rose from 26.8 to 33.8%, with the duration of breastfeeding showing tandem increases (Leung *et al*, 2002b). However, the impact of these recent changes would not yet have occurred.

Epidemiological studies (Collaborative Group on Hormonal Factors in Breast Cancer, 1996) have implicated long-term exposure to oral contraceptives as a risk factor for premenopausal breast cancer. However, recent age-period-cohort modelling papers have failed to find such an effect on the ecological level (Persson *et al*, 1993, 1998). Moreover, women in Hong Kong used oral contraceptives much less frequently than their Western counterparts. For instance, regular surveys by the Family Planning Association of Hong Kong found that of all women who practised contraception generally, oral pill users decreased from 27 to 16% from 1982 to 1997 (The Family Planning Association of Hong Kong, 1999). Given the small magnitude of the relative risk increase associated with long-term pill use and breast cancer development (Collaborative Group on Hormonal Factors in Breast Cancer, 1996), the potential excess absolute risk in the local population would be minimal, if present. We observed a statistically significant inflection point in the 1910 birth cohort and associated increased risk ratios for women born during 1910–1915 compared to neighbouring birth cohorts (Table 4 and Figure 3). We believe this to be a real change given the magnitude of effect and the decreasing linear trend from 1910 through 1925. This may be related to breast development and hormonal changes during puberty of these women who started adolescence during 1920–1925, the period between the First World War and the Great Depression. In consequence, these women would have experienced a relatively prosperous environment during adolescence in terms of nutrition and lifestyle generally compared to those born immediately before and after them. Other researchers have documented similar findings in Norway (Tretli and Gaard, 1996) and Shanghai (Jin *et al*, 1993). We also found that there was a nadir in breast cancer rates in the 1930 birth cohort, after which there was a sustained rise. Similar to our previous observation for the 1910–1915 cohorts, we believe this indicates lifestyle factors that changed among adolescent women during the Second World War influenced their risk of breast cancer. Previous research (Tretli and Gaard *et al*, 1996) suggested dietary patterns and physical activity level during this crucial period of development contributed to the observed results.

Two important messages can be derived from our findings. First, it is important to take into account birth cohort patterns in incidence analyses for breast cancer when exploring the underlying factors responsible for secular trends. Birth cohort effects must also be considered when monitoring and evaluating the effects of early detection, treatment and intervention programmes using population disease rates. Second, the three main putative factors of fertility, diet, and an affluent lifestyle manifesting as cohort effects in our results can all be thought of as direct or indirect consequences of westernisation. As China moves towards the western way of life, through globalisation and economic development, we can expect an emerging epidemic of breast cancer as Hong Kong's experience demonstrated. Even Hong Kong is only beginning to see the crest of this wave of breast cancer cases, 20 years after the rest of the Western world. China will probably experience similar rises in secular trends as Hong Kong in another 15 to 20 years, assuming similar rates of westernisation and consistencies in age-period-cohort effects.

## References

[bib1] ClaytonDSchifflersE1987a Models for temporal variation in cancer rates, I: age-period and age-cohort models Stat Med 6 449467 3629047 10.1002/sim.4780060405

[bib2] ClaytonDSchifflersE1987b Models for temporal variation in cancer rates, II: age-period and age-cohort models Stat Med 6 469481 3629048 10.1002/sim.4780060406

[bib3] Collaborative Group on Hormonal Factors in Breast Cancer1996 Breast cancer and hormonal contraceptives: collaborative reanalysis of individual data on 53 297 women with breast cancer and 100 239 women without breast cancer from 54 epidemiological studies Lancet 347 17131727 8656904 10.1016/s0140-6736(96)90806-5

[bib4] Collaborative Group on Hormonal Factors in Breast Cancer2002 Breast cancer and breastfeeding: collaborative reanalysis of individual data from 47 epidemiological studies in 30 countries, including 50 302 women with breast cancer and 96 973 women without the disease Lancet 360 187195 12133652 10.1016/S0140-6736(02)09454-0

[bib5] HolfordTR1991 Understanding the effects of age, period, and cohort on incidence and mortality rates Ann Rev Public Health 12 425457 2049144 10.1146/annurev.pu.12.050191.002233

[bib6] HolfordTR1992 Analysing the effects of age, period, and cohort on incidence and mortality rates Stat Meth Med Res 1 317337 10.1177/096228029200100306 1341663

[bib7] HolfordTRRoushGCMcKayLA1991 Trends in female breast cancer in Connecticut and the United States J Clin Epidemiol 44 2939 1986055 10.1016/0895-4356(91)90198-i

[bib8] Hong Kong Cancer Registry1999 Cancer incidence and mortality in Hong Kong 1995–1996.Hospital Authority: Hong Kong

[bib9] JinFShuXODevesaSSZhengWBlotWJGaoYT1993 Incidence trends for cancers of the breast, ovary, and corpus uteri in urban Shanghai, 1972–1989 Cancer Causes Control 4 355360 8347785 10.1007/BF00051338

[bib10] LeungGMLamTHThachTQHedleyAJ2002a Will screening mammography in the East do more harm than good? Am J Public Health(Scheduled to be published November, 2002) 10.2105/ajph.92.11.1841 PMC1447338 12406818

[bib11] LeungGMHoLMLamTH2002b Breastfeeding rates in Hong Kong: a comparison of the 1987 and 1997 birth cohorts Birth 29 162168 12153646 10.1046/j.1523-536x.2002.00183.x

[bib12] MadiganMPZieglerRGBenichouJByrneCHooverRN1995 Proportion of breast cancer cases in the United States explained by well-established risk factors J Nat Cancer Inst 87 16811685 7473816 10.1093/jnci/87.22.1681

[bib13] McCullaghPNelderJ1983 Generalized linear models,2nd ednLondon: Chapman and Hall

[bib14] MoradiTAdamiHOBergstromRGrindleyGWolkAGerhardssonMDosemeciMNyrenO1999 Occupational physical activity and risk for breast cancer in a nationwide cohort study in Sweden Cancer Causes Control 10 423430 10530613 10.1023/a:1008922205665

[bib15] ParkinDMWhelanSLFerlayJRaymondLYoungJ(eds)1997 Cancer incidence in five continents.Vol. VIIIARC Scientific Publications No. 143.Lyon, France: International Agency for Research on Cancer, World Health Organisation

[bib16] PerssonIBergstromRBarlowLAdamiHO1998 Recent trends in breast cancer incidence in Sweden Br J Cancer 77 167169 9459163 10.1038/bjc.1998.26 PMC2151267

[bib17] PerssonIBergstromRSparenPThornMAdamiHO1993 Trends in breast cancer incidence in Sweden 1958–1988 by time period and birth cohort Br J Cancer 68 12471253 8260381 10.1038/bjc.1993.513 PMC1968654

[bib18] RostgaardKVaethMHolstHMadsenMLyngeE2001 Age-period-cohort modelling of breast cancer incidence in the Nordic countries Stat Med 20 4761 11135347 10.1002/1097-0258(20010115)20:1<47::aid-sim613>3.0.co;2-5

[bib19] SeowADuffySWMcGeeMALeeJLeeHP1996 Breast cancer in Singapore: trends in incidence 1968–1992 Int J Epidemiol 25 4045 8666502 10.1093/ije/25.1.40

[bib20] ShahparCLiG1999 Homicide mortality in the United States, 1935–1994: age, period, and cohort effects Am J Epidemiol 150 12131222 10588082 10.1093/oxfordjournals.aje.a009948

[bib21] TaroneREChuK1992 Implications of birth cohort patterns in interpreting trends in breast cancer rates J Natl Cancer Inst 84 14021410 1512791 10.1093/jnci/84.18.1402

[bib22] TaroneREChuK1996 Evaluation of birth cohort patterns in population disease rates Am J Epidemiol 143 8591 8533751 10.1093/oxfordjournals.aje.a008661

[bib23] The Family Planning Association of Hong Kong1981, 1986, 1991, 1996, 2001 Youth Sexuality Study Reports in 1981, 1986, 1991, 1996 and 2001

[bib24] The Family Planning Association of Hong Kong1999 Report on the survey of family planning in Hong Kong 1997: knowledge, attitude, practice,Hong Kong: The Family Planning Association of Hong Kong

[bib25] TretliSGaardM1996 Lifestyle changes during adolescence and risk of breast cancer: an ecologic study of the effect of World War II in Norway Cancer Causes Control 7 507512 8877047 10.1007/BF00051882

[bib26] WunLPFeuerEJMillerBA1995 Are increases in mammographic screening still a valid explanation for trends in breast cancer incidence in the United States? Cancer Causes Control 6 135144 7749053 10.1007/BF00052774

[bib27] YipPSFLeeJChanBAuJ2001 A study of demographic changes under sustained below-replacement fertility in Hong Kong SAR Soc Sci Med 53 10031009 11556770 10.1016/s0277-9536(00)00395-6

[bib28] YuHHarrisREGaoYTGaoRWynderEL1991 Comparative epidemiology of cancers of the colon, rectum, prostate and breast in Shanghai, China versus the United States Int J Epidemiol 20 7681 2066247 10.1093/ije/20.1.76

