# Trends in breast cancer incidence in Hong Kong between 1973 and 1999: an age-period-cohort analysis

**DOI:** 10.1038/sj.bjc.6601019

**Published:** 2003-06-10

**Authors:** G M Leung, T Q Thach, T-H Lam, A J Hedley, W Foo, R Fielding, P S F Yip, E M C Lau, C-M Wong

**Correction to:** *British Journal of Cancer* (2002), **87**, 982–988. doi:10.1038/sj.bjc.6600583

The average annual percentage change (AAPC) as quoted in the paper currently says 3.61%. This figure is actually for the average *triennial* percentage change. Similarly, all the corresponding age-specific AAPCs in Table 2 are average triennial percentage change.

The authors apologise for any inconvenience this may have caused.

